# The First Mitochondrial Genome for Geastrales (*Sphaerobolus stellatus*) Reveals Intron Dynamics and Large-Scale Gene Rearrangements of Basidiomycota

**DOI:** 10.3389/fmicb.2020.01970

**Published:** 2020-08-11

**Authors:** Jinghua Ye, Jie Cheng, Yuanhang Ren, Wenlong Liao, Qiang Li

**Affiliations:** ^1^College of Information Science & Technology, Chengdu University, Chengdu, China; ^2^College of Food and Biological Engineering, Chengdu University, Chengdu, China

**Keywords:** mitochondrial genome, protein coding gene, repeat sequence, intron, gene rearrangement, phylogenetic analysis

## Abstract

In this study, the mitogenome of artillery fungus, *Sphaerobolus stellatus*, was assembled and compared with other Basidiomycota mitogenomes. The *Sphaerobolus stellatus* mitogenome was composed of circular DNA molecules, with a total size of 152,722 bp. Accumulation of intergenic and intronic sequences contributed to the *Sphaerobolus stellatus* mitogenome becoming the fourth largest mitogenome among Basidiomycota. We detected large-scale gene rearrangements in Basidiomycota mitogenomes, and the *Sphaerobolus stellatus* mitogenome contains a unique gene order. The quantity and position classes of intron varied between 75 Basidiomycota species we tested, indicating frequent intron loss/gain events occurred in the evolution of Basidiomycota. A novel intron position classes (P1281) was detected in the *Sphaerobolus stellatus* mitogenome, without any homologous introns from other Basidiomycota species. A pair of fragments with a total length of 9.12 kb in both the nuclear and mitochondrial genomes of *Sphaerobolus stellatus* was detected, indicating possible gene transferring events. Phylogenetic analysis based on the combined mitochondrial gene set obtained well-supported tree topologies (Bayesian posterior probabilities ≥ 0.99; bootstrap values ≥98). This study served as the first report on the mitogenome from the order Geastrales, which will promote the understanding of the phylogeny, population genetics, and evolution of the artillery fungus, *Sphaerobolus stellatus*.

## Introduction

The genus *Sphaerobolus* belongs to the family Sphaerobolaceae and the order Geastrales. It is widely distributed globally, and most commonly found on wood mulches (Geml et al., 2005b). The *Sphaerobolus* species is called artillery fungus because it can eject a 1-mm diameter “gleba” (spore mass) up to 6 m toward the brightest light in its environment (Ingold, 1968, 1969). Since the first documentation of *Sphaerobolus* nearly 300 years ago, many mycologists have focused on investigations of the growth and reproduction of the fungus (Geml et al., 2005a). According to morphological characteristics, the *Sphaerobolus* genus has been classified as a member of the class Gasteromycetes along with other fungi having passive spore discharge, including puffballs, earth balls, and earth stars. Molecular data assigned the genus *Sphaerobolus* into the gomphoid-phalloid clade with *Phallus*, *Ramaria*, and *Gomphus* as closest relatives (Geml et al., 2005a, b). Geml et al. (2005b) have classified the *Sphaerobolus* genus into three recognized species, including *S. stellatus*, *S. iowensis*, and *S. ingoldii*, based on molecular data and morphological characteristics. *S. stellatus* was the first reported and representative species of the genus *Sphaerobolus*. Several nuclear molecular markers and mitochondrial sequences have been used for the classification and identification of *Sphaerobolus* species, including the internal transcribed spacer regions of the nuclear ribosomal gene repeat (ITS), nuclear large ribosomal RNA subunit (LSU), translation elongation factor 1-a gene (EF 1-α), and mitochondrial ribosomal RNA small subunit (mtSSU) (Geml et al., 2005a, b). However, up till now, there is no complete mitochondrial genome (mitogenome) from the genus *Sphaerobolus*, or even from the order Geastrales has been reported, which limits understanding of the origin and evolution of this unique artillery fungus.

Mitochondria are important organelles of eukaryotes, which are the main sources of energy for the growth and development of eukaryotes (Ernster and Schatz, 1981; McBride et al., 2006). Mitochondria contain their own genomes, which are believed to have originated from Alphaproteobacteria by the ancestors of eukaryotes through endosymbiosis (Munoz-Gomez et al., 2017). Mitogenome has been widely used to analyze the evolution and phylogeny of eukaryotes due to the characteristics of uniparental inheritance, rapid evolution rate and several available molecular markers (Richards et al., 1998; Li et al., 2015, Li et al., 2020c). However, the current understanding of the mitogenome characteristics of Basidiomycota is limited, mainly due to the limited number of complete mitogenomes available. The available Basidiomycota mitogenomes (<120) were far less than the animal mitogenomes (>9,400) published, or even the number of Basidiomycota nuclear genomes (>5,700) reported^1^. The phylum Basidiomycota is the largest group of mushroom-forming fungi on earth. Analysis of Basidiomycota mitogenomes will help us to understand the origin and evolution of mushrooms (Li et al., 2020a). Previous studies have shown that the evolution rate of fungal mitogenome was intermediate between animals (the highest) and plants (the lowest) (Aguileta et al., 2014). In addition, the genome size, gene content, base composition, intron number, repeat sequences and gene arrangement varied greatly in the mitogenome of fungi (Mardanov et al., 2014; Li et al., 2018a; Sandor et al., 2018). However, most Basidiomycota mitogenomes contained a set of protein coding genes (PCGs), including *atp6*, *atp8*, *atp9*, *cob*, *cox1*, *cox2*, *cox3*, *nad1*, *nad2*, *nad3*, *nad4*, *nad4L*, *nad5*, and *nad6* for energy metabolism and *rps3* for transcriptional regulation, which we called core PCGs of Basidiomycota (Li et al., 2018b, 2019a, 2020b).

In the present study, the mitogenome of representative species from the genus *Sphaerobolus*, *S. stellatus*, was assembled, annotated and compared with other basidiomycete mitogenomes. The aims of this study are: (1) to reveal the characterization of the *S. stellatus* mitogenome; (2) to reveal the variations or similarities between *S. stellatus* and other Basidiomycota mitogenomes in genome size, base composition, gene content and gene arrangement by comparative mitogenomic analysis; (3) to reveal the intron dynamics of *cox1* genes in Basidiomycota mitogenomes; (4) to understand the phylogenetic status of *S. stellatus* in the phylum Basidiomycota based on the combined mitochondrial gene set. This study served as the first report on the mitogenome from the order Geastrales, which will promote the understanding of the origin, evolution and genetics of Geastrales species.

## Materials and Methods

### Mitogenome Assembly and Annotations

The raw sequencing data of *S. stellatus* used for mitogenome assembly were downloaded from the Sequence Read Archive (SRA) (acc. SRR3928187) (Kohler et al., 2015). The raw sequencing data were firstly passed through a series of quality control steps to generate clean reads, which included filtering low-quality sequences and removing adapter reads by using AdapterRemoval v 2 (Schubert et al., 2016). The mitogenome of *S. stellatus* was *de novo* assembled using SPAdes 3.9.0 software with a kmer size of 17 (Bankevich et al., 2012). Gaps between contigs obtained were filled using MITObim V1.9 (Hahn et al., 2013), as well as separate PCR and Sanger sequencing. Then, we obtained a circular mitochondrial genome for *S. stellatus*. We annotated the complete mitogenome of *S. stellatus* according to our previously described methods (Li et al., 2018a, c). Briefly, the protein-coding genes (PCGs), rRNA genes, tRNA genes, and introns of the *S. stellatus* mitogenome were initially annotated using MITOS (Bernt et al., 2013) and MFannot (Valach et al., 2014), both based on the genetic code 4 (the Mold, Protozoan, and Coelenterate Mitochondrial Code). This was followed by modification and prediction of PCGs using the NCBI Open Reading Frame Finder (NCBI Resource Coordinators, 2017) under the genetic code 4, and further annotation by BLASTP searches against the NCBI non-redundant protein sequence database (Bleasby and Wootton, 1990). Intron-exon borders of PCGs were verified using the exonerate v2.2 software (Slater and Birney, 2005). The tRNA genes in the *S. stellatus* mitogenome were also predicted with tRNAscan-SE v1.3.1 (Lowe and Chan, 2016). Graphical map of the *S. stellatus* mitogenome was drawn with OGDraw v1.2 (Lohse et al., 2013).

### Sequence and Repetitive Element Analyses of the *S. stellatus* Mitogenome

Base compositions of the *S. stellatus* mitogenome and other Basidiomycota mitogenomes were calculated using the DNASTAR Lasergene v7.1^2^. We calculated strand asymmetries of the Basidiomycota mitogenomes according to the following formulas: AT skew = [A – T]/[A + T], and GC skew = [G – C]/[G + C] (Wang et al., 2017). BLASTN searches (Chen et al., 2015) of the *S. stellatus* mitogenome against itself were conducted to identify any intra-genomic duplications of large fragments or interspersed repeats throughout the *S. stellatus* mitogenome, using an *E*-value of <10^–10^ as a threshold. Tandem Repeats Finder (Benson, 1999) was used to detect tandem repeats (>10 bp) within the *S. stellatus* mitogenome. Repeated sequences in the *S. stellatus* mitogenome were also detected by REPuter to identify forward (direct), reverse, complemented, and palindromic (revere complemented) repeats (Kurtz et al., 2001). We also conducted BLASTN searches of the mitogenome against its published nuclear genome (CVRD00000000.1) to identify if there was natural gene segments transferred between the *S. stellatus* nuclear genome and its mitogenome.

### Comparative Mitogenomic Analysis and Intron Analysis

To assess conservations and variations between the reported Basidiomycota mitogenomes, we conducted comparative mitogenomic analyses of mitogenome sizes, GC content, base composition, gene and intron numbers, and gene arrangement. Introns of *cox1* genes of the 75 Basidiomycota mitogenomes tested were classified into different position classes (Pcls) according to previously described methods (Ferandon et al., 2010). The *cox1* genes of 75 Basidiomycota species were first aligned with the *cox1* gene of the medical fungus, *Ganoderma calidophilum*, by Clustal W (Thompson et al., 1994), which served as the reference (Li et al., 2019d). The Pcls were named according to its insertion site in the corresponding reference sequence. The same Pcls from different species were considered homologous and usually had high sequence similarities (Ferandon et al., 2010).

### Phylogenetic Analysis

The phylogenetic status of the *S. stellatus* mitogenome within the phylum *Basidiomycota* was analyzed based on the combined mitochondrial gene set (15 core PCGs + 2 rRNA genes) (Li et al., 2018c). A total of 75 *Basidiomycota* species was included in the phylogenetic analysis, and *Annulohypoxylon stygium* from the phylum Ascomycota was used as the outgroup (Deng et al., 2018). We first aligned individual mitochondrial genes using the MAFFT v7.037 software (Katoh et al., 2017), and then concatenated the aligned mitochondrial genes into a combined mitochondrial gene set using the SequenceMatrix v1.7.8 (Vaidya et al., 2011). Potential phylogenetic conflicts between different mitochondrial genes were detected using a preliminary partition homogeneity test. The best-fit models of evolution and partitioning schemes for the mitochondrial gene set was determined using PartitionFinder 2.1.1 (Lanfear et al., 2017). Both Bayesian inference (BI) and maximum likelihood (ML) methods were used to construct the phylogenetic tree. RAxML v 8.0.0 (Stamatakis, 2014) was used to perform the ML analysis. We used MrBayes v3.2.6 (Ronquist et al., 2012) to conduct the BI analysis. Two independent runs with four chains (three heated and one cold) each were conducted simultaneously for 2 × 10^6^ generations. Each run was sampled every 100 generations. We assumed that stationarity had been reached when the estimated sample size (ESS) was greater than 100, and the potential scale reduction factor (PSRF) approached 1.0. The first 25% samples were discarded as burn-in, and the remaining trees were used to calculate Bayesian posterior probabilities (BPP) in a 50% majority-rule consensus tree.

## Results

### Characterization and PCGs of the *S. stellatus* Mitogenome

**FIGURE 1:**
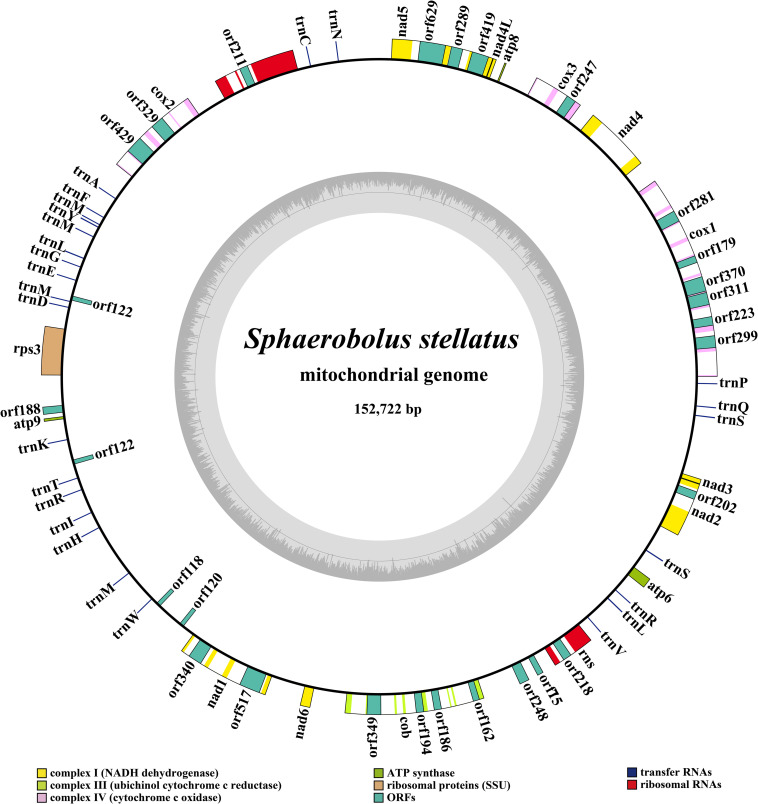
Circular map of the mitochondrial genome of *Sphaerobolus stellatus*. Genes are represented by different colored blocks. Colored blocks outside each ring indicate that the genes are on the direct strand, while colored blocks within the ring indicates that the genes are located on the reverse strand.

The complete mitogenome of *S. stellatus* was composed of circular DNA molecules with a total size of 152,722 bp (Figure 1). The GC content of the *S. stellatus* mitogenome was 27.05%. The mitogenome of *S. stellatus* had negative AT skew and GC skew (Supplementary Table S1). The *S. stellatus* mitogenome was found containing 43 PCGs, of which 22 belonged to non-intronic PCGs and the other 21 located in introns (Supplementary Table S2). The 22 non-intronic PCGs included a whole set of core PCGs (*atp6*, *atp8*, *atp9*, *cob*, *cox1*, *cox2*, *cox3*, *nad1*, *nad2*, *nad3*, *nad4*, *nad4L*, *nad5*, *nad6*, and *rps3*) and 7 non-conserved PCGs. Non-conserved PCGs in the *S. stellatus* mitogenome mainly encoded proteins with unknown functions. A total of 34 introns were detected in the mitogenome of *S. stellatus*, 32 of which belonged to the group I (Supplementary Table S2). The 21 intronic ORFs in the *S. stellatus* mitogenome included 14 ORFs encoding LAGLIDADG endonuclease, 6 ORFs encoding GIY-YIG endonuclease, and 1 ORFs with unknown functions.

### rRNA and tRNA Genes

**FIGURE 2:**
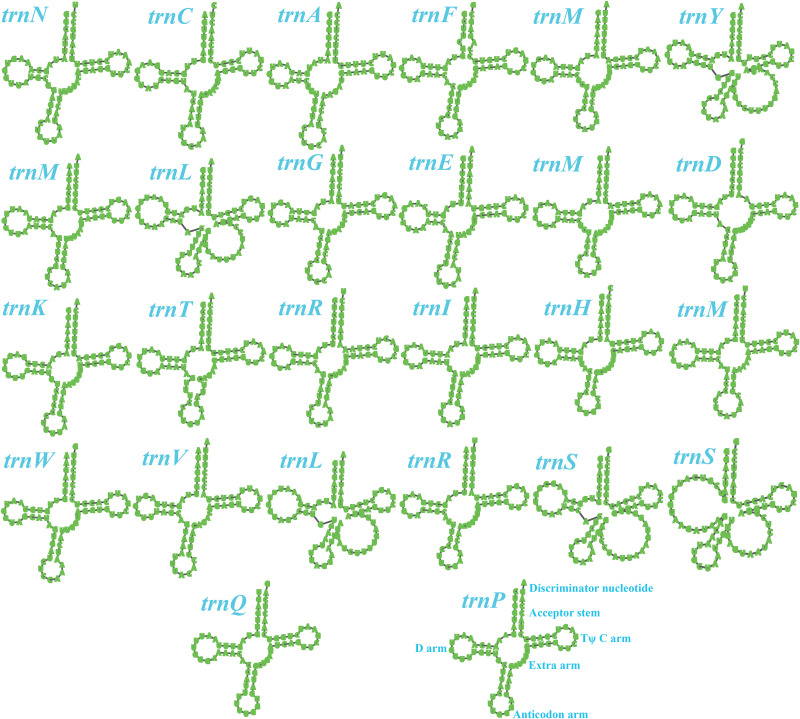
Putative secondary structures of the 26 tRNA genes identified in the mitochondrial genome of *Sphaerobolus stellatus*. All genes are shown in order of occurrence in the mitochondrial genome of *Sphaerobolus stellatus*, starting from *trnN.*

Two rRNA genes were detected in the *S. stellatus* mitogenome, including the large subunit ribosomal RNA (*rnl*) and the small subunit ribosomal RNA (*rns*) (Supplementary Table S2). The mitogenome of *S. stellatus* contained 26 tRNA genes, which were folded into classical cloverleaf structures (Figure 2). The mitogenome of *S. stellatus* contained 2 tRNAs with different anticodons coding for leucine, arginine and serine, 4 tRNAs with the same anticodons coding for methionine. The length variations of extra arms contributed to the size variations of tRNA genes, with each ranging from 71 to 87 bp. Of all the 71 genes detected in the *S. stellatus* mitogenome, 67 genes were located on the direct strand and the other 4 genes were on the reverse strand.

### Mitogenome Compositions and Codon Usage

**FIGURE 3:**
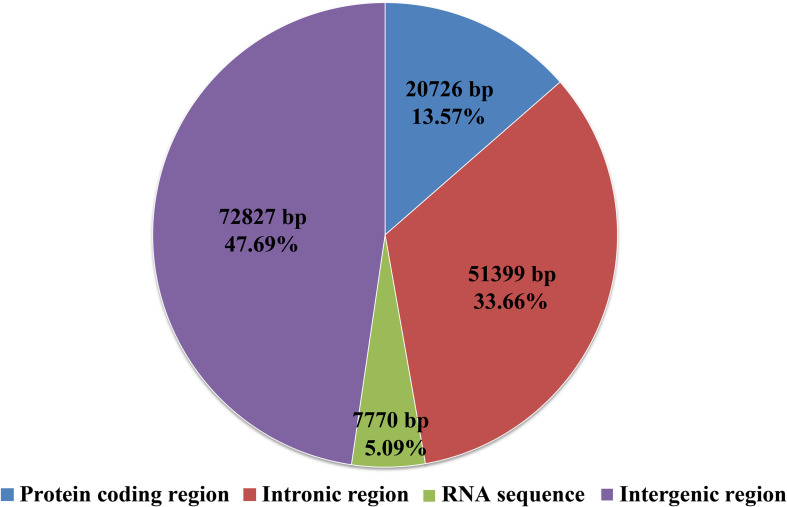
The protein-coding, intronic, intergenic, and RNA gene region proportions of the entire mitochondrial genome of *Sphaerobolus stellatus*.

Intergenic region was the largest among all the regions in the *S. stellatus* mitogenome, which accounted for 47.69% of the entire mitogenome (Figure 3). The result indicated that the mitogenome of *S. stellatus* had a loose structure. A total of 51,399 bp of intronic sequences were detected in the mitogenome of *S. stellatus*, which comprised 33.66% of the *S. stellatus* mitogenome. Protein coding regions was the third largest part of the *S. stellatus* mitogenome, accounting for 13.57% of the entire mitogenome. RNA coding region accounted for 5.08% of the *S. stellatus* mitogenome.

**FIGURE 4:**
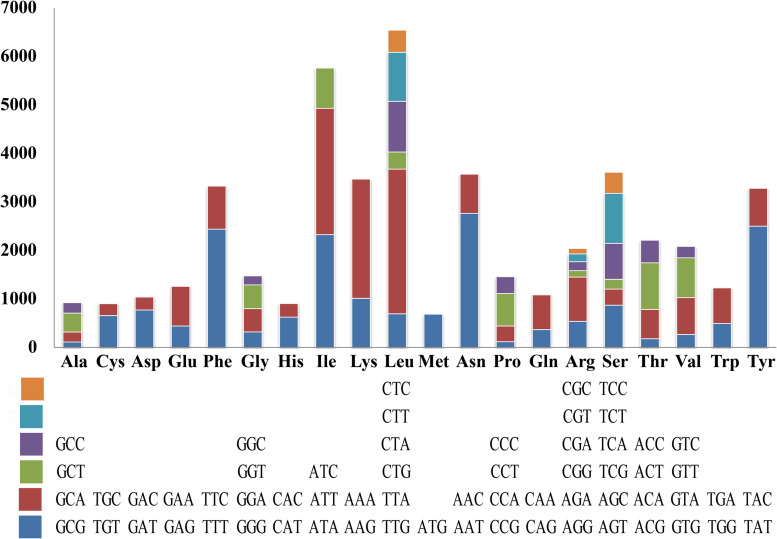
Codon usage in the mitochondrial genome of *Sphaerobolus stellatus*. Frequency of codon usage is plotted on the y-axis.

Codon usage analysis indicated that the most frequently used codons in the *S. stellatus* mitogenome were TTA (for leucine; Leu), AAT (for asparagine; Asn), ATT (for isoleucine; Ile), TAT (for tyrosine; Tyr) and AAA (for lysine; Lys) (Figure 4). The high frequency of A and T used in codons contributed to the high AT content of the *S. stellatus* mitogenome (72.95%) (Supplementary Table S3).

### Repeat Elements in the *S. stellatus* Mitogenome

BLASTN searches of the *S. stellatus* mitogenome against itself identified 95 repetitive sequences in the *S. stellatus* mitogenome (Supplementary Table S4). The length of these repetitive sequences ranged from 28 bp to 101 bp, with pair-wise nucleotide similarities ranging from 87.76% to 100%. The longest repetitive sequences were detected in the coding region of ORF157. A total of 3,823 bp of repetitive sequences were detected in the mitogenome of *S. stellatus*, which accounted for 2.50% of the entire mitogenome.

A total of 329 tandem repeats were detected in the mitogenome of *S. stellatus*, which accounted for 2.95% of the entire mitogenome (Supplementary Table S5). The longest tandem sequence was found between the neighboring genes *cox2* and *trnA*, with a size of 55 bp. Most tandem repeat sequences in the mitogenome of *S. stellatus* were copied 2 - 5 times, with the highest copy number of 26. We also identified 32 forward, 2 palindromic and 16 reverse repeats in the mitogenome of *S. stellatus* though REPuter (Kurtz et al., 2001) (Supplementary Table S6), which accounted for 2.24% of the whole mitogenome.

To identify if there were gene segments that transferred between the nuclear and mitochondrial genomes, we blasted the *S. stellatus* mitogenome against its nuclear genome. A total of 53 aligned fragments were detected in the mitogenome of *S. stellatus*, with each aligned fragment ranging from 34 bp to 1,468 bp (Supplementary Table S7). The sequence identities of these aligned fragments were between 89.13% and 100%. The largest aligned fragment was found located in the intergenic region between *trnG* and *orf122*, and encompassed the coding region of *trnE*. The presence of large fragments aligned between the nuclear and mitochondrial genomes of the *S. stellatus* mitogenome indicated that genetic transfer between nuclear and mitochondrial genome may have occurred in the evolution of *S. stellatus*.

### Intron Dynamics of *cox1* Genes in Basidiomycota

**FIGURE 5:**
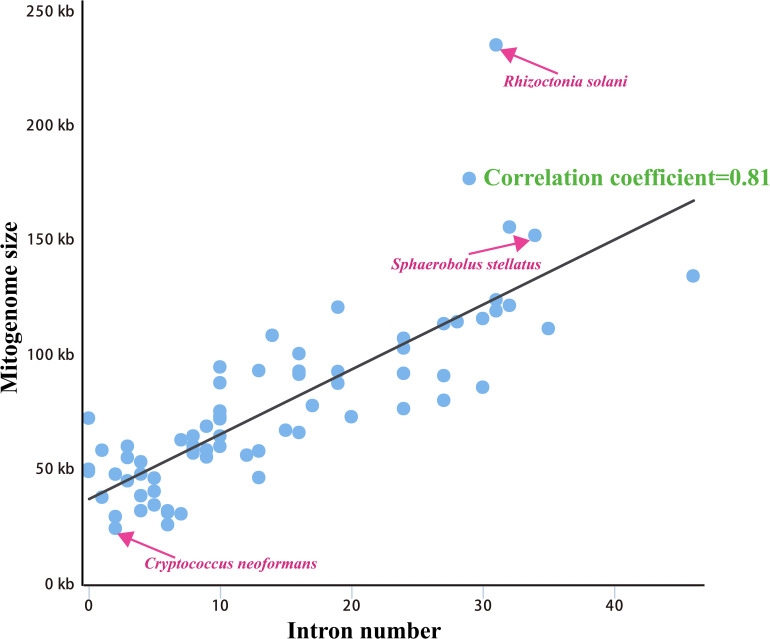
Pearson correlation analysis between mitogenome sizes and intron numbers of 75 Basidiomycota species.

We calculated correlations between mitogenome sizes and intron numbers of the 75 Basidiomycota species. The results showed that the number of intron was closely related to the mitogenome size of Basidiomycota, with the pearson correlation coefficient of 0.81 (Figure 5). Therefore, the dynamics of intron could significantly promote the size variations of Basidiomycota mitogenomes. According to the insertion position of introns in the coding region of host genes, we could classify fungal introns into different position classes (Pcls). In the present study, a total of 1046 introns were detected in the 75 Basidiomycota mitogenomes, with each Basidiomycota species containing 0 – 46 mitochondrial introns. Large variations in intron number indicated that intron gain/loss events have occurred in the evolution of Basidiomycota species. These introns were harbored in 14 host genes: *atp6*, *atp9*, *cob*, *cox1*, *cox2*, *cox3*, *nad1*, *nad2*, *nad3*, *nad4*, *nad4L*, *nad5*, *rns*, and *rnl* genes.

**FIGURE 6:**
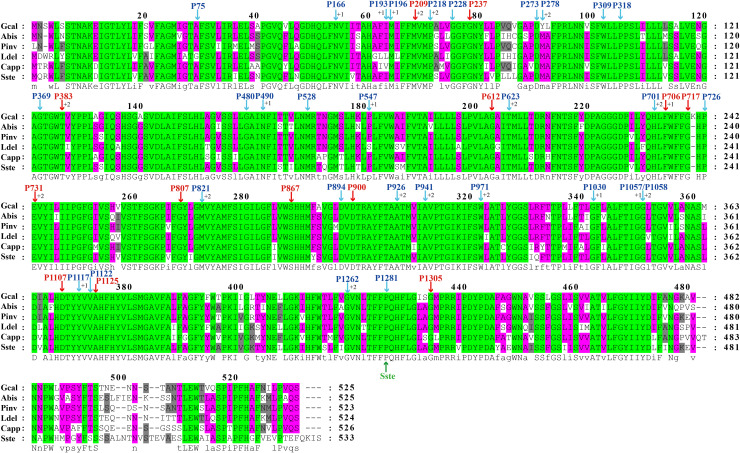
The insertion sites of different Pcls in *cox1* coding regions of 6 basidiomycete species from different orders. The *cox1* amino acids of the 6 basidiomycete species were first aligned using Clustal W. The Pcls in red indicate that they are widely distributed introns in the 75 basidiomycetes, while Pcls in blue indicate they are rare Pcls in the 75 basidiomycetes. The symbol ‘Sste’ in green font represents the novel intron identified in *Sphaerobolus stellatus*. The symbols ‘+1’ and ‘+2’ indicate that the insertion of the intron occurs inside the indicated codon: between the nt 1 and nt 2 of this codon for ‘+1’ and between the nt 2 and nt 3 for ‘+2’. Species ID are shown in Supplementary Table S1.

**FIGURE 7:**
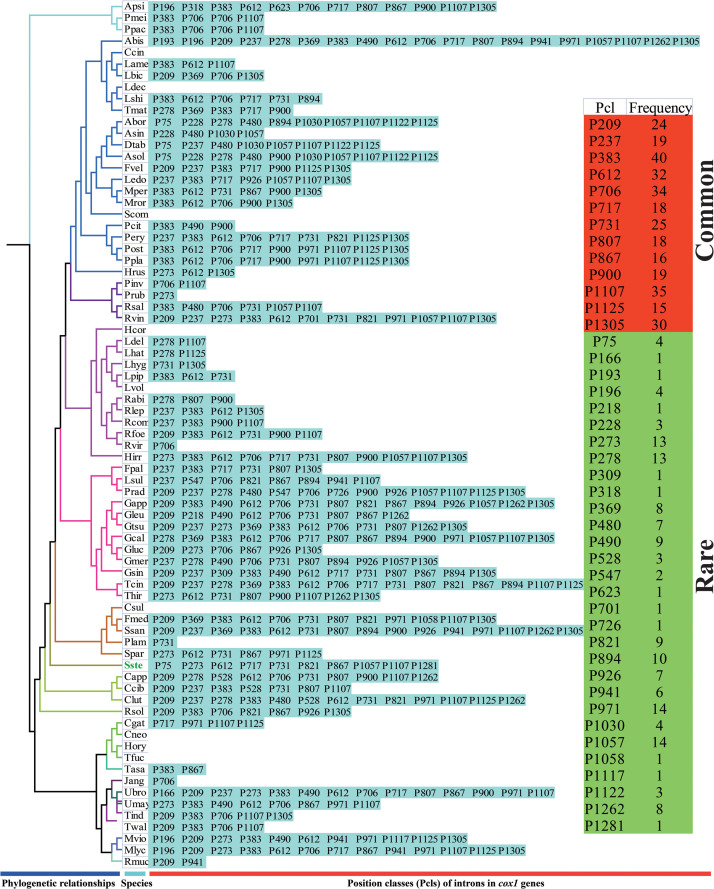
Pcl information of *cox1* gene of the 75 *Basidiomycota* species. The Pcls were name according to its insertion site in the corresponding reference sequence (*Ganoderma calidophilum*: MH252535). Introns present in more than 1/5 Basidiomycota species was considered as common introns. The phylogenetic positions of the 75 Basidiomycota species were established using the Bayesian inference (BI) method and Maximum Likelihood (ML) method based on 15 concatenated mitochondrial core proteins and 2 rRNA genes. Species ID are shown in Supplementary Table S1.

The largest host gene of Basidiomycetes introns was the *cox1* gene, which harbored 33.93% of the mitochondrial introns. We analyzed the intron dynamics of *cox1* gene in the present study. A total of 43 Pcls were detected in *cox1* genes of the 75 Basidiomycota species, of which 13 were considered as widely distributed Pcls (present in more than 1/5 Basidiomycota species) (Figure 6). P383 was the most common Pcl in Basidiomycota mitogenomes, which was distributed in 40 of the 75 Basidiomycota species (Figure 7). Followed by the P1107, it could be detected in 35 of the 75 Basidiomycota mitogenomes. Thirty Pcls were considered as rare Pcls in Basidiomycota, which were distributed in less than 1/5 Basidiomycota species. Several Pcls, including P166, P193, P218, P309, P318, P623, P701, P726, P1058, P1117, and P1281, were only distributed in one of the 75 Basidiomycota species. However, some of these rare Pcls were detected in mitogenomes from distant species, such as *Chaetosphaeridium globosum* (Turmel et al., 2002) and *Rhizophydium* sp. 136 (Forget et al., 2002), indicating possible horizontal gene transfer events occurred in evolution. The mitogenome of *S. stellatus* contained 1 rare Pcls (P1281) never detected in other species, indicating high diversities of introns in the *S. stellatus* mitogenome. The origin and evolution of introns in *S. stellatus* and other Basidiomycota species needs further studies.

### Comparative Mitogenome and Gene Arrangement Analyses

The sizes of 75 Basidiomycota mitogenomes we tested varied greatly, ranging from 24,874 bp to 235,849 bp, with an average size of 77,184 bp (Supplementary Table S1). The 152,722 bp mitogenome of *S. stellatus* was the fourth largest among the 75 Basidiomycota mitogenomes detected, which was only smaller than *Phlebia radiata* (156, 348 bp) (Salavirta et al., 2014) from the order Polyporales, *Rhizoctonia solani* (235,849 bp) (Losada et al., 2014) from the order Cantharellales, and *Ustilago bromivora* (177,540 bp) (acc. LT558140 in the NCBI database) from the order Ustilaginales. The GC content of the *S. stellatus* mitogenome was about the average (27.34%) of that of the 75 Basidiomycota species. Forty-one of the 75 Basidiomycota mitogenomes had negative AT skews, and the remaining 34 had positive AT skews. Among the 75 Basidiomycota mitogenomes we detected, 57 species had positive GC skews. The mitogenome of *Rhizoctonia solani* contained the most PCGs, with each Basidiomycota species containing 14-127 PCGs. The *S. stellatus* mitogenome contained the third most introns among the 75 Basidiomycota mitogenomes detected, which was only less than *Agaricus bisporus* (Ferandon et al., 2013) and *Sanghuangporus sanghuang* (Han et al., 2018). All the 75 Basidiomycota species contained two rRNA genes. In addition, 20–35 tRNA genes were detected in the 75 Basidiomycota species.

**FIGURE 8:**
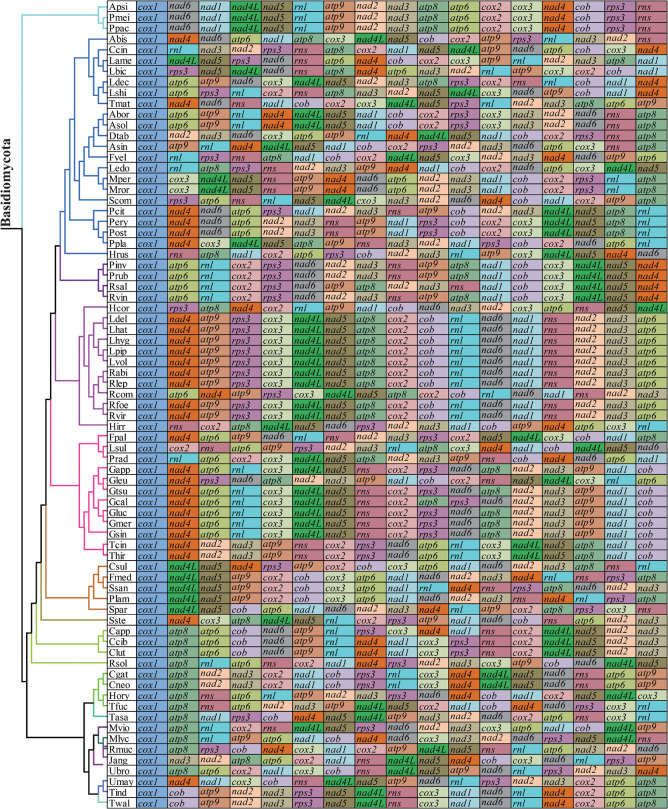
Mitochondrial gene arrangement analysis of 75 Basidiomycota mitogenomes. 15 core protein coding genes and 2 rRNA genes were included in the gene arrangement, starting from *cox1* gene.

The arrangements of 15 core PCGs and 2 rRNA genes varied greatly at family levels (Figure 8). Any species from different families had different gene orders, indicating large-scale gene rearrangements occurred in the evolution of Basidiomycota mitogenomes. Even within the same genera, we also observed large-scale gene rearrangements, including *Laccaria*, *Rhizopogon*, *Lyophylum*, *Armillaria*, *Ustilago*, and *Microbotryum*. Mitochondrial gene shifts and inversions were observed in the mitogenome of *S. stellatus* compared with other mitogenomes, which showed the *S. stellatus* mitogenome had a unique gene order among all Basidiomycota species detected.

### Phylogenetic Analysis

**FIGURE 9:**
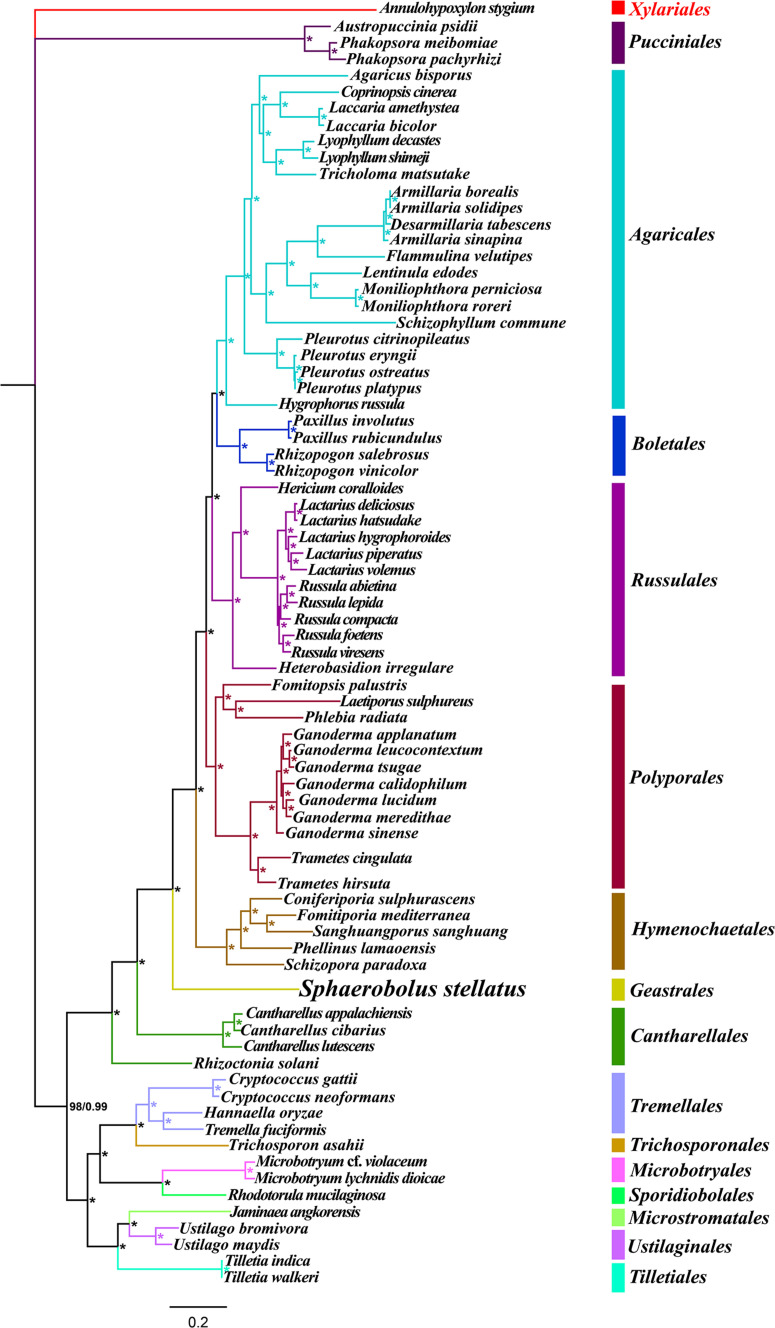
Molecular phylogeny of 75 Basidiomycota species based on Bayesian inference (BI) and Maximum Likelihood (ML) analyses of 15 protein coding genes and two rRNA genes. Support values are bayesian posterior probabilities (BPP, before slash) and bootstrap values (BS, after slash). The asterisk indicates that the BPP and BS values are 1 and 100, respectively. Species and NCBI accession numbers for genomes used in the phylogenetic analysis are provided in Supplementary Table S1.

Identical and well-supported tree topologies were obtained using maximum likelihood (ML) and Bayesian inference (BI) methods based on the combined mitochondrial gene set (15 core PCGs + 2 rRNA genes) (Figure 9). All major clades within the trees had good support values (BPP ≥ 0.99; BS ≥ 98). Based on the phylogenetic analysis, the 75 *Basidiomycota* species could be divided into 15 major clades, corresponding to the orders *Pucciniales*, *Agaricales*, *Boletales*, *Russulales*, *Polyporales, Hymenochaetale*s, *Geastrales, Cantharellales*, *Tremellales*, *Trichosporonales*, *Microbotryales*, *Sporidiobolales*, *Microstromatales*, *Ustilaginales*, and *Tilletiales*. The phylogenetic analysis indicated that *S. stellatus* had close relationships with *Hymenochaetale*s and *Cantharellales*. The results showed that mitochondrial genes were effective molecular markers for phylogenetic analysis of *Basidiomycota* species.

## Discussion

### Size Variations and Intron Dynamics of Basidiomycota Mitogenomes

Compared with conservative mitogenome sizes of animals, fungal mitogenome sizes varied greatly. The mitogenome size of 75 Basidiomycota species we tested ranged from 24,874 bp to 235,849 bp. The mitogenome of *S. stellatus* was the fourth largest in Basidiomycota. Correlation analysis showed that the number of introns was closely related to the size of mitogenomes in Basidiomycota. The results indicated that the variation of introns was the main factor leading to the size variations of mitogenomes in Basidiomycota, which was consistent with previous studies (Mardanov et al., 2014; Liu et al., 2019). In some mitogenomes, such as *Rhizoctonia solani*, *Phlebia radiata*, and *S. stellatus*, we found accumulation of intergenic sequences and plasmid-derived genes (Losada et al., 2014; Salavirta et al., 2014), which also led to the size variations of mitogenomes. The *cox1* gene was the largest host gene of basidiomycete introns (Wang et al., 2020), which harbored 33.93% of the total inrons in the 75 basidiomycetes. So the dynamics of introns in *cox1* genes could markedly affect mitogenome size of basidiomycetes. In addition, the exon-intron borders of rRNA genes in Basidiomycota are difficult to identify accurately. So we analyzed the intron dynamics of *cox1* gene in the present study. We found that the quantity and Pcl of intron varied greatly between different Basidiomycota species, even between species from the same genera, which indicated that intron loss/gain events occurred in the evolution of Basidiomycota. However, some rare Pcls in Basidiomycota were detected from distinct species in other phyla (Forget et al., 2002; Turmel et al., 2002), showing potential horizontal gene transferring events. The *S. stellatus* mitogenome contained a novel intron, which was never detected from other species. Further studies are needed to reveal the origin and evolution of introns in *S. stellatus*.

### Gene Rearrangements in Basidiomycota Mitogenomes

In the present study, we found that Basidiomycota species from different families had different mitochondrial gene arrangements, indicating large-scale gene rearrangements occurred in the evolution of Basidiomycota (Li et al., 2019b). Compared with the mitogenome of Basidiomycota, the arrangement of mitochondrial genes in animals is more conservative (Aguileta et al., 2014). However, with the rapid development of next generation sequencing, mitochondrial genome rearrangements were also detected in some animal species and several models were proposed to reveal rearrangements of mitogenomes in animals (Sankoff et al., 1992; Lavrov et al., 2002; Xia et al., 2016). The mechanism of mitogenome rearrangement in fungi was less studied and the accumulation of repetitive sequences was believed to be closely related to the rearrangement of fungal mitogenomes (Aguileta et al., 2014). The average repeat content of the 75 Basidiomycota mitogenomes was >2.5%, which may lead to gene rearrangements in Basidiomycota mitogenomes. In addition, we found that the *S. stellatus* mitogenome had a unique gene arrangement, which was different from other species in Basidiomycota. More mitogenomes from the order Geastrales need to be sequenced and analyzed to assess conservations and variations of mitochondrial gene arrangement in the order Geastrales.

### Gene Transfer Between Mitochondrial and Nuclear Genomes

Most mitochondrial genes have been transferred to nuclear genomes in evolution, which was considered to have many advantages (Adams and Palmer, 2003). So far, only a dozen to one hundred mitochondrial genes have been retained in eukaryotic mitogenomes (Allen, 2015). Genes naturally transferring between nuclear and mitochondrial genome have been observed in various organisms, which plays an important role in species evolution and environmental adaptation (Adams et al., 2002; Zhao et al., 2018). In the present study, large aligned fragments between the mitochondrial and nuclear genomes of *S. stellatus* were observed, which included gene coding regions and intergenic regions. The effects of natural gene transferring between mitochondrial and nuclear genomes on the evolution and development or growth of *S. stellatus* need to be further studied.

### Phylogenetic Analysis of Basidiomycota

Limited morphological characters and the overlapping of some morphological features make it difficult to identify Basidiomycota species accurately (Li et al., 2019c). With the rapid development of next generation sequencing technology, mitochondrial genes have been widely used as molecular markers to analyze population genetics, taxonomy and biogeography of animals (Cameron, 2014; Li et al., 2015; Wang and Xu, 2020; Wang et al., 2020). However, phylogenetic studies of Basidiomycota species based on combined mitochondrial gene sets were few due to limited number of fungal mitogenomes available in public databases. In the present study, over two thirds of Basidiomycota mitogenomes available were included in the phylogenetic study. A well-supported phylogenetic tree was obtained based on the combined mitochondrial gene set. The result indicted that the mitogenome was suitable for study of phylogeny of Basidiomycota species. More mitogenomes of Basidiomycota need to be studied to reveal the evolution and phylogeny of Basidiomycetes.

## Data Availability Statement

The datasets presented in this study can be found in online repositories. The names of the repository/repositories and accession number(s) can be found in the article/Supplementary Material.

## Author Contributions

QL, YR, and JY conceived and designed the experiments and contributed reagents, materials, and analysis tools. QL and JC performed the experiments. QL, WL, and JC analyzed the data. QL wrote the manuscript. All authors contributed to the article and approved the submitted version.

## Conflict of Interest

The authors declare that the research was conducted in the absence of any commercial or financial relationships that could be construed as a potential conflict of interest.

## Footnotes

1 https://www.ncbi.nlm.nih.gov/genome/browse#!/overview/

2 http://www.dnastar.com/
